# Unusual “Mini-Rugby Ball” Pattern Solitary Lung Metastasis in Relapsed Ewing's Sarcoma

**DOI:** 10.1055/s-0044-1788793

**Published:** 2024-08-06

**Authors:** Abhay Gondhane, Sunita N. Sonavane, Sandip Basu

**Affiliations:** 1 Radiation Medicine Centre, Bhabha Atomic Research Centre, Tata Memorial Hospital Annexe, Jerbai Wadia Road, Parel, Mumbai, Maharashtra, India; 2 Homi Bhabha National Institute, Mumbai, Maharashtra, India

**Keywords:** Ewing's sarcoma, ^18^ F-NaF, ^18^ F-FDG, PET/CT, lung metastases

## Abstract

Ewing's sarcoma (ES) is a mesenchymal origin malignant neoplasm that affects children and adolescents. It is the second most common type of bone sarcoma and accounts for approximately 1.5% of all childhood cancers with an annual incidence of 1 to 3 cases per million children under 16 years of age. In this article, we present the case of a 16-year-old adolescent girl. Lung metastasis at the initial diagnosis of ES is relatively uncommon but carries significant prognostic implications. Lung metastases in ES can vary significantly in size, ranging from small nodules (just a few millimeters in size) to the largest reported case being 15 cm. The size of the metastases impacts the choice of therapeutic strategies and the prognosis. Approximately 30% of patients with ES experience a relapse, with the lungs being a common site for metastatic disease. Relapsed lung metastasis on follow-up is a critical concern in the long-term management of ES. We describe a relapsed case of ES in a 16-year-old adolescent girl who presented with a solitary large metastatic right lung mass, with the longest dimension of 16 cm on craniocaudal measurement. The primary site of the tumor was the left distal femur, for which the patient received six cycles of neoadjuvant chemotherapy, followed by en bloc tumor excision and rotationplasty of the left distal femur, after which the patient received seven cycles of adjuvant chemotherapy. Subsequent 5 years of regular follow-up was asymptomatic. Later, the patient presented with back pain and cough, and was diagnosed with a solitary large right lung mass. Computed tomography (CT) guided biopsy of the right lung mass revealed a metastatic ES, for which she underwent chemoradiotherapy. This case highlights the large size of solitary lung metastases in relapsed ES.

## Introduction

Ewing's sarcoma (ES) was described for the first time in 1921 by James Stephen Ewing, American histopathologist, oncologist, and hematologist. 1 ES is a mesenchymal origin malignant neoplasm that affects children and adolescents. Its peak incidence is in the second decade of life, with the average age being 13 to 16 years. 2 It is the second most common type of bone sarcoma and it accounts for approximately 1.5% of all childhood cancers with an annual incidence is 1 to 3 cases per million children under 15 years of age. 3 Clinically it manifests as intermittent bone pain at the site of the primary bone involvement, which increases in intensity at night. Sometimes, pain is associated with fever, weight loss, and anorexia. 4 The most affected bone sites include metaphysis of the long bones (around 56%), costal arches (∼15–17%), flat bones (16%), and skull (3–4%). 5 The size of the primary ES tumor at presentation can vary widely, typically ranging from 5 to 10 cm in diameter. 5 The incidence of lung metastases at the primary presentation of ES ranges from 10 to 25%. 6 The most common route of metastases in ES is hematogenous, causing the lung, bone, bone marrow, and brain metastases. However, the lymphatic route is found less frequently. 7 Lung metastases in ES can vary significantly in size, from small nodules to large masses exceeding 10 cm in diameter. 8 Diagnosis of ES includes radiological imaging studies like X-rays of the primary site, computed tomography (CT), and magnetic resonance imaging (MRI). A definite diagnosis is by the histological evaluation of primary bone lesion. Histopathologically, the presence of a series of chromosomal translocations that culminate in the fusion of the *EWSR1* gene on chromosome 22 with one of several members of the erythroblast transformation specific (ETS) family of transcription factors is the defining characteristic of these tumors. The most common of these translocations includes t (11;22) (q24;q12), which fuses the *EWSR1* gene with the *FLI1* gene on chromosome 11 and is present in approximately 90% of cases. 9 Treatment includes extensive surgery of the lesion with free section margins followed by chemotherapy and radiotherapy. 10 Chemotherapy can be before (neoadjuvant) or after surgery (adjuvant). The most commonly used regimens worldwide are VACA (vincristine, actinomycin, cyclophosphamide, doxorubicin) and VAC/IE (vincristine, cyclophosphamide, doxorubicin alternating with ifosfamide/etoposide). 9 The prognosis depends on age, clinical stage at diagnosis, and presence of metastases at the time of diagnosis. 10 Patients diagnosed early in the initial stages of the disease have a better survival. Mortality is high, especially in the first year after diagnosis in patients with lung metastases. 11

## Case Report

**Fig. 1:**
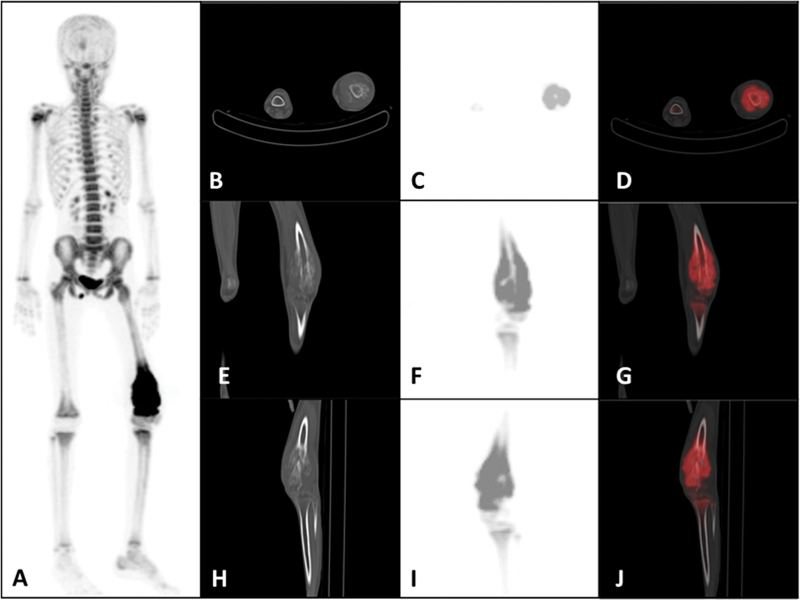
( **A** ) Anterior maximum intensity projection (MIP) of whole-body ^18^ F-sodium fluoride ( ^18^ F-NaF) positron emission tomography (PET). Noncontrast computed tomography (CT; bone window), PET, and fused PET/CT: ( **B–D** ) axial, ( **E–G** ) coronal, and ( **H–J** ) sagittal images showing increased tracer uptake noted in the site of the primary bone tumor involving the left distal femur (maximum standardized uptake value [SUV _max_ ] measuring 10.98).

**Fig. 2:**
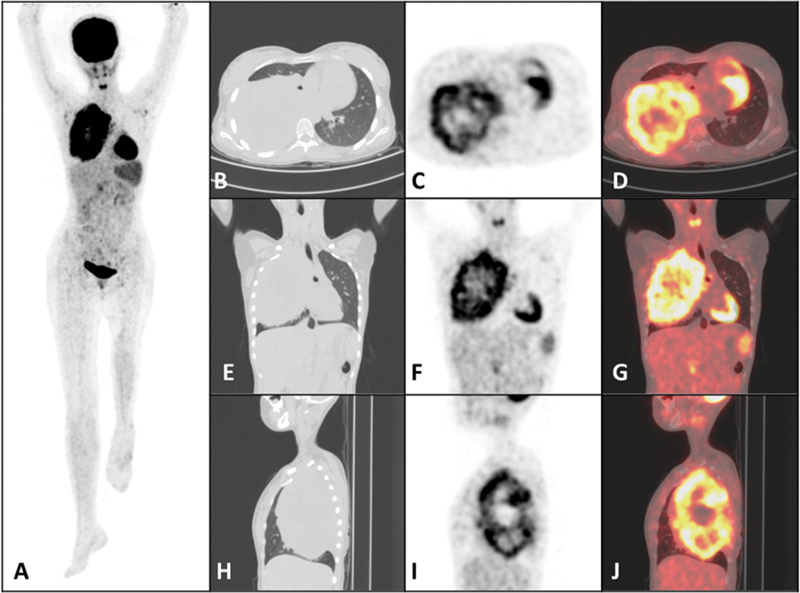
( **A** ) Anterior maximum intensity projection of whole-body ^18^ F-fluorodeoxyglucose positron emission tomography ( ^18^ F-FDG PET). Noncontrast computed tomography (CT; lung window), PET, and fused PET/CT: ( **B–D** ) axial, ( **E–G** ) coronal, and ( **H–J** ) sagittal images showing metabolically active heterogenous soft-tissue lesion with central necrotic area involving almost a significant area of the right lung parenchyma, measuring 10.7 × 11.2 × 16 cm (maximum standardized uptake value [SUV _max_ ] of 8.05).

A 16-year-old adolescent girl presented with on and off episodes of fever, pain, and swelling over the distal part of the left thigh and knee joint. Left knee MRI showed altered marrow signal, appearing heterogeneously hyperintense on short tau inversion recovery (STIR) images and hypointense on T1-weighted images, noted in the distal diaphysis and metaphysis on the femur reaching up to the growth plate. Mild marrow edema in the distal epiphysis was noted as hyperintensity on STIR images. Periosteal reaction was noted with the presence of circumferential abnormal soft-tissue component measuring 7.6 × 6.4 cm in the transverse plane and 1.8 cm along the long axis. ^18^ Fluorine-sodium fluoride ( ^18^ F-NaF) positron emission tomography/CT (PET/CT) scan was performed 60 minutes after intravenous injection of 9.43 mCi of ^18^ F-NaF, using a whole-body full ring dedicated 3D PET/CT scanner covering from the vertex to the toe region. Whole-body noncontrast CT (100 mA, 120 kV, 2 mm) was acquired for attenuation correction and anatomical localization. Images were reconstructed using the standard iterative algorithm (RAMLA). Images were reformatted into transaxial, coronal and sagittal views, which showed the solitary site of increased tracer uptake (maximum standardized uptake value [SUV _max_ ] of 10.98) in the left distal femur lesion ( Fig. 1 ). No lung nodules, skip lesion, or any other site of skeletal abnormality were noted. Fine-needle aspiration cytology from the left distal femur lesion revealed small, monomorphic round cells with fine nuclear chromatin, irregularly vacuolated cytoplasm (periodic acid–Schiff [PAS] positive) and round nuclei, most likely ES. The patient received six cycles of neoadjuvant chemotherapy VIDE (vincristine, ifosfamide, doxorubicin, etoposide), which was followed by wide excision of the left distal femur lesion and limb salvage surgery, that is, rotational plasty of the left distal femur. Subsequently, the patient further received seven cycles of adjuvant chemotherapy VAI (vincristine, dactinomycin, ifosfamide), and VAC (vincristine, dactinomycin, cyclophosphamide). The patient was asymptomatic and was on regular follow-up for the subsequent 5 years. On follow-up, she presented with complaints of back pain and cough. Chest X-ray showed a large homogenous area of consolidation in the right lung field. Thus, a whole-body fluorine-18-fluorodeoxyglucose ( ^18^ F-FDG) PET/CT scan was performed by a similar protocol as the previous PET scan after intravenous injection of 5.6 mCi of ^18^ F-FDG. Scan findings revealed no abnormal tracer uptake at the site of the primary, with postoperative status and evidence of rotationplasty noted over the left distal femur. A solitary large hypermetabolic (SUV _max_ of 8.05) heterogenous soft-tissue lesion with central necrotic area was noted involving almost a significant area of the right lung parenchyma, measuring 10.7 × 11.2 × 16 cm (anteroposterior [AP] × transverse [T] × craniocaudal [CC]), almost the size of a mini-rugby ball ( Fig. 2 ). Further CT-guided biopsy of the right lung mass revealed a metastatic ES. Subsequently, in view of the relapsed ES presenting as pulmonary mass, the patient received eight cycles of salvage chemotherapy with the VTC (vincristine, topotecan, cyclophosphamide) regimen. Postchemotherapy CT scan of the chest showed a decrease in the size of the solitary metastatic lesion in the right lung, measuring 7.1 × 9.5 × 13 cm (AP × T × CC). The rest of the lung parenchyma was unremarkable. In view of the significant size of the residual lesion, the patient received external beam radiotherapy (EBRT), 55.8 Gy in 31 fractions to the right lung mass. The patient is on follow-up and is stable, under observation at present. The presented case highlights an unusual large-sized solitary lung metastasis in a relapsed patient of ES.

## Discussion

ES is a rare and aggressive malignancy that primarily affects children and young adults. 12 Karski et al 13 and Ramkumar et al 14 reported that advanced age may increase the probability of metastasis of ES. Our patient was diagnosed at 16 years of age and at the time of relapse and diagnosis of lung metastases, she was 23 years of age. Primary ES of the lung is exceedingly rare, with only 17 cases identified in the literature. 15 Thomas et al reported thoracic ES in an 18-year-old man. 16 The incidence of lung metastases at the primary presentation of ES ranges from 10 to 25%. 6 17 A primary tumor size more than 8 cm has an increased likelihood of having metastatic diseases at initial diagnosis. 18 The presentation of lung metastases at the initial diagnosis of ES is relatively uncommon, but carries significant prognostic implications. 19 This early dissemination indicates a more aggressive disease course and necessitates comprehensive staging at diagnosis. 20 The main treatment modality for the primary ES lesion is surgery with en bloc resection of the tumor and nearby soft tissues or amputation of the limb; in particular cases, limb salvage surgery is proposed. 1 2 In Sanchez-Saba et al's 9 study of 88 patients of ES of bone treated with preoperative chemotherapy and limb-sparing surgery, the overall survival rates were 79.5% at 2 years, 69% at 5 years, and 64% at 10 years. According to them, limb-sparing surgery associated with pre- and postoperative chemotherapy should be the treatment for ES of bone that meets certain requirements that allow its performance.

During treatment, lung metastases can be detected using imaging modalities such as CT and PET scans. 21 The detection of new metastases during therapy often requires a modification of the treatment plan, including possible escalation of chemotherapy or consideration of surgical interventions. 22 Studies have shown that approximately 30% of patients with ES experience a relapse, with the lungs being a common site for metastatic disease. 23 24 The largest lung metastases from ES reported in the literature measured over 15 cm. 25 The size of the metastases can impact the choice of therapeutic strategies and the prognosis. 26 Such extensive disease poses significant therapeutic challenges and often requires multimodal treatment approaches. 26 Lung metastases in ES are relatively rare compared with other sites of metastases such as bones and bone marrow. 27 Novel diagnostic features include the use of molecular imaging and liquid biopsies to detect circulating tumor cells and DNA. 28 Large lung metastases present significant therapeutic challenges, including the difficulty of achieving complete surgical resection and the limited efficacy of radiation therapy for bulky disease. 29 Chemotherapy remains a mainstay treatment, but high-dose regimens are often required. 30 There are ongoing controversies in the treatment of lung metastases in ES, particularly regarding the role of surgical resection versus nonsurgical approaches. 5 Some studies advocate for aggressive surgical management, while others suggest that systemic therapy alone may be sufficient in certain cases. 31 Management dilemmas include deciding on the timing and extent of surgical intervention, the use of adjuvant therapies, and balancing treatment efficacy with quality-of-life considerations. 32 Multidisciplinary teams are essential in navigating these complex decisions to optimize patient outcomes. 22 At relapse for the solitary large metastatic lung lesion of ES, in the present case, the patient was given chemotherapy and external radiotherapy. However, there was persistent residual disease and necessitated further treatment. We highlight the rarity in view of the large size of the metastatic lung lesion.

## References

[1] Ortiz Escobar A Monsalvo Zuleta H Martínez Rangel D Ewing's sarcoma in a public children's hospital in Cartagena: case series, 2010–2012 Scientific Spike 2014 11 87 92

[2] Ewing J Classics in oncology. Diffuse endothelioma of bone. James Ewing. Proceedings of the New York Pathological Society, 1921 CA Cancer J Clin 1972 22 02 95 98 4622125 10.3322/canjclin.22.2.95

[3] Izaguirre G A Mejía K I Castro C A Ewing's sarcoma with pulmonary metastases in a pediatric patient: case report Archives of Medicine MedPub Journals 2016 12 02 5

[4] Jiménez S D Soto F J Garro O M Vega U G Ewing's sarcoma Rev ClE Med UCR 2014 4 9 12

[5] Hawkins D S Paulino A C Doyle J Ewing sarcoma of the pelvis: long-term survival and prognostic factors Pediatr Blood Cancer 2004 42 06 567 573

[6] Carvajal R Meyers P Ewing's sarcoma and primitive neuroectodermal family of tumors Hematol Oncol Clin North Am 2005 19 03 501 525, vi–vii 15939194 10.1016/j.hoc.2005.03.004

[7] Hernandez E H Mosquera C G Quintero M O Hernandez C I Ewing's sarcoma Magazine Medical Archive of Camagüey 2013 17 623 640

[8] Miser J S Goldsby R E Chen Z Treatment of metastatic Ewing sarcoma/primitive neuroectodermal tumor of bone: evaluation of increasing the dose intensity of chemotherapy: a report from the Children's Oncology Group Pediatr Blood Cancer 2007 49 07 894 900 17584910 10.1002/pbc.21233

[9] Sanchez-Saba J E Abrego M O Albergo J I Sarcoma de Ewing óseo. Enfoque multidisciplinario y resultados oncológicos en 88 pacientes. [Ewing sarcoma of the bone. Multidisciplinary approach and oncological results in 88 patients] Medicina (B Aires) 2020 80 01 23 30 32044738

[10] Gaspar N Hawkins D S Dirksen U Ewing sarcoma: current management and future approaches through collaboration J Clin Oncol 2015 33 27 3036 3046 26304893 10.1200/JCO.2014.59.5256

[11] Bosma S E Ayu O Fiocco M Gelderblom H Dijkstra P DS Prognostic factors for survival in Ewing sarcoma: a systematic review Surg Oncol 2018 27 04 603 610 30449479 10.1016/j.suronc.2018.07.016

[12] Ladenstein R Pötschger U Le Deley M C Primary disseminated multifocal Ewing sarcoma: results of the Euro-EWING 99 trial J Clin Oncol 2010 28 20 3284 3291 20547982 10.1200/JCO.2009.22.9864

[13] Karski E E Matthay K K Neuhaus J M Goldsby R E Dubois S G Characteristics and outcomes of patients with Ewing sarcoma over 40 years of age at diagnosis Cancer Epidemiol 2013 37 01 29 33 22959474 10.1016/j.canep.2012.08.006 PMC3543501

[14] Ramkumar D B Ramkumar N Miller B J Henderson E R Risk factors for detectable metastatic disease at presentation in Ewing sarcoma: an analysis of the SEER registry Cancer Epidemiol 2018 57 134 139 30412903 10.1016/j.canep.2018.10.013

[15] Deokar K K Kunjir N G Ghorpade S Primary Ewings sarcoma of the lung J Clin Diagn Res 2015 9 01 XD01 XD03 10.7860/JCDR/2015/10946.5436 PMC4347161 25738070

[16] Thomas A Obeidat N Darweesh M Thoracic Ewing's sarcoma: a case report Cureus 2022 14 04 e24150 35592195 10.7759/cureus.24150 PMC9110040

[17] Cotterill S J Ahrens S Paulussen M Prognostic factors in Ewing's tumor of bone: analysis of 975 patients from the European Intergroup Cooperative Ewing's Sarcoma Study Group J Clin Oncol 2000 18 17 3108 3114 10963639 10.1200/JCO.2000.18.17.3108

[18] Ye C Dai M Zhang B Risk factors for metastasis at initial diagnosis with Ewing sarcoma Front Oncol 2019 9 1043 31681581 10.3389/fonc.2019.01043 PMC6805828

[19] Grier H E Krailo M D Tarbell N J Addition of ifosfamide and etoposide to standard chemotherapy for Ewing's sarcoma and primitive neuroectodermal tumor of bone N Engl J Med 2003 348 08 694 701 12594313 10.1056/NEJMoa020890

[20] Grünewald T GP Cidre-Aranaz F Surdez D Ewing sarcoma Nat Rev Dis Primers 2018 4 01 5 29977059 10.1038/s41572-018-0003-x

[21] Khoury J D Navarro S Epstein A L Circulating tumor DNA analysis in Ewing sarcoma family of tumors Mol Cancer Ther 2018 17 06 1337 1346

[22] Liu T Li Y Li X Diagnosis and treatment of lung metastases in Ewing's sarcoma Oncol Lett 2020 20 03 2725 2733

[23] Children's Oncology Group Leavey P J Mascarenhas L Marina N Prognostic factors for patients with Ewing sarcoma (EWS) at first recurrence following multi-modality therapy: a report from the Children's Oncology Group Pediatr Blood Cancer 2008 51 03 334 338 18506764 10.1002/pbc.21618 PMC2728357

[24] Marina N Anderson J R Kauh J Assessment of early response to chemotherapy and overall survival in patients with metastatic Ewing's sarcoma J Clin Oncol 2014 32 03 303 310

[25] Saif M W Rahman A Ewing's sarcoma: updates on management and future directions J Oncol Pract 2014 10 04 288 295

[26] Jürgens H Exner U Gadner H Multidisciplinary treatment of primary Ewing's sarcoma of bone. A 6-year experience of a European Cooperative Trial Cancer 1988 61 01 23 32 3334950 10.1002/1097-0142(19880101)61:1<23::aid-cncr2820610106>3.0.co;2-m

[27] Esiashvili N Goodman M Marcus R B Jr Changes in incidence and survival of Ewing sarcoma patients over the past 3 decades: Surveillance Epidemiology and End Results data J Pediatr Hematol Oncol 2008 30 06 425 430 18525458 10.1097/MPH.0b013e31816e22f3

[28] Duchman K R Lynch C F Buckwalter J A Ewing sarcoma in the United States: epidemiology, prognosis, and treatment outcome J Surg Oncol 2015 111 07 877 885

[29] Womer R B West D C Krailo M D Randomized controlled trial of interval-compressed chemotherapy for the treatment of localized Ewing sarcoma: a report from the Children's Oncology Group J Clin Oncol 2012 30 33 4148 4154 23091096 10.1200/JCO.2011.41.5703 PMC3494838

[30] European Intergroup Cooperative Ewing's Sarcoma Study-92 Paulussen M Craft A W Lewis I Results of the EICESS-92 Study: two randomized trials of Ewing's sarcoma treatment: cyclophosphamide compared with ifosfamide in standard-risk patients and assessment of benefit of etoposide added to standard treatment in high-risk patients J Clin Oncol 2008 26 27 4385 4393 18802150 10.1200/JCO.2008.16.5720

[31] Applebaum M A Goldsby R Neuhaus J DuBois S G Clinical features and outcomes in patients with Ewing sarcoma and regional lymph node involvement Pediatr Blood Cancer 2012 59 04 617 620 22184129 10.1002/pbc.24053 PMC3310932

[32] Ferrari S Palmerini E The treatment of Ewing's sarcoma: current status and outlook Rev Recent Clin Trials 2007 2 01 89 101

